# Further analysis of previously implicated linkage regions for Alzheimer's disease in affected relative pairs

**DOI:** 10.1186/1471-2350-10-122

**Published:** 2009-12-01

**Authors:** Elin S Blom, Vilmantas Giedraitis, Sampath Arepalli, Marian L Hamshere, Omanma Adighibe, Alison Goate, Julie Williams, Lars Lannfelt, John Hardy, Fabienne Wavrant-De Vrièze, Anna Glaser

**Affiliations:** 1 Section of Molecular Geriatrics, Department of Public Health and Caring Sciences, Uppsala University, Uppsala, Sweden; 2 Laboratory of Neurogenetics, National Institute of Aging, National Institute of Health, Bethesda, MD, USA; 3 Department of Psychological Medicine & Biostatistics and Bioinformatics Unit, Wales School of Medicine, Cardiff University, Cardiff, UK; 4 Department of Psychiatry, Washington University School of Medicine, St. Louis, MO, USA

## Abstract

**Background:**

Genome-wide linkage studies for Alzheimer's disease have implicated several chromosomal regions as potential loci for susceptibility genes.

**Methods:**

In the present study, we have combined a selection of affected relative pairs (ARPs) from the UK and the USA included in a previous linkage study by Myers *et al*. (Am J Med Genet, 2002), with ARPs from Sweden and Washington University. In this total sample collection of 397 ARPs, we have analyzed linkage to chromosomes 1, 9, 10, 12, 19 and 21, implicated in the previous scan.

**Results:**

The analysis revealed that linkage to chromosome 19q13 close to the *APOE *locus increased considerably as compared to the earlier scan. However, linkage to chromosome 10q21, which provided the strongest linkage in the previous scan could not be detected.

**Conclusion:**

The present investigation provides yet further evidence that 19q13 is the only chromosomal region consistently linked to Alzheimer's disease.

## Background

Alzheimer's disease (AD) is the most common form of dementia and the number of affected individuals rises dramatically with an aging population. Age is the most prominent risk factor, but genetics is also important for the risk of developing AD. Three genes are known to cause autosomal dominant early-onset AD: the amyloid beta precursor protein (*APP*) on chromosome 21 [1], presenilin 1 (*PSEN1*) on chromosome 14 [2] and presenilin 2 (*PSEN2*) on chromosome 1 [3]. For the much more common sporadic AD with later onset, apolipoprotein E (*APOE*) on chromosome 19q13 is so far the only identified susceptibility gene with consistently demonstrated association [4].

The *ε4 *allele of *APOE *is estimated to account for less than a third of the lifetime risk for AD [5,6] and simulation studies have predicted at least four additional genetic loci contributing to age at onset [7]. Although such calculations are by necessity based on certain assumptions, they support the possibility that there are more genetic susceptibility factors for AD to be identified. Genome-wide linkage studies using affected sib-pairs or families have implicated a number of chromosomal loci to hold susceptibility genes [8-14]. Regions on chromosomes 9, 10, 12 and 19 seem to be the most replicated, although the exact position of the peaks can differ substantially.

In the present study, we have combined a selection of affected relative pairs (ARPs) from the UK and the USA included in an earlier linkage study by Myers *et al*. [10]. We have modified the original sample collection by excluding the NIMH sample and samples with ambiguous phenotypes, as well as by adding sample collections from Sweden and Washington University. We have analyzed linkage to regions on chromosomes 1, 9, 10, 12, 19 and 21, previously implicated in the study by Myers *et al*.

## Methods

### Samples

A total of 580 individuals from 261 families affected by late onset AD (family mean age at onset ≥60 years) divided into 397 ARPs were analyzed in this study. Out of these, 116 ARPs were collected in Sweden, 87 ARPs in the UK and 194 ARPs in the USA (Indiana Alzheimer Disease Center National Cell Repository and Washington University, St. Louis, MO) (Table 1).

**Table 1:** Sample information

Sample	PED	IND	AIND	ASP	ACP	*APOE ε4+*	*APOE ε4-*	AAO ± SD
SWE	52	168	130	102	14	87	10	69.1 ± 6.2
UK	70	148	148	87	0	51	16	75.0 ± 5.9
USA	139	369	302	191*	3	143	16	72.6 ± 6.1
Total	261	685	580	380	17	281	42	72.4 ± 5.5

PED: number of pedigrees, IND: number of genotyped individuals, AIND: number of genotyped affected individuals, ASP: number of genotyped affected sib-pairs, ACP: number of genotyped pairs of affected first cousins, *APOE ε4+*: number of ARPs where both individuals have at least one *APOE ε4 *allele, *APOE ε4-*: number of ARPs where neither individual has an *APOE ε4 *allele, AAO: age at onset, SD: standard deviation. * Including one half-sib pair.

The ARPs were selected from families where at least one relative pair was diagnosed with possible, probable or definite AD according to NINCDS-ADRDA diagnostic criteria [15]. All available family members, including unaffected relatives, were sampled and genotyped after informed consent had been collected from each participating individual or next of kin. Only Caucasian families were included to reduce potential genetic heterogeneity. This study was approved by local and national ethics committees.

Samples from the UK and the Indiana Alzheimer Disease Center National Cell Repository were also included in the study by Myers *et al*. To improve power of the present study, samples with ambiguous phenotypes were removed and new samples were added. This resulted in a total of 244 affected individuals from the UK and USA samples (129 ARPs) that were also genotyped in the study by Myers *et al*. but with another microsatellite marker set. Twelve of the families from Sweden were analyzed in Giedraitis *et al*. 2006 [16]. There is also a likely overlap with the Swedish samples used in the present study and the sample collection used by Sillén *et al*. [13,14], but the extent of this overlap is unknown to us.

### Genotyping

A total of 100 microsatellite markers on chromosomes 1, 9, 10, 12, 19 and 21 also used in a study by Blacker *et al*. [8] were included. The markers had an average spacing of 9.4 cM and an average genotyping success rate of 86% (Table 2 and Additional file 1). In addition, *APOE *was included as a genetic marker. Data from an additional 170 microsatellite markers located on other chromosomes and with an average genotyping success rate of <80% were included in the analysis of family structure, but not in the linkage analysis.

**Table 2:** Microsatellite marker information

Chromosome	Number of microsatellite markers	Average intermarker distance (cM)	Genotyping success rate (%)
1	31	8.9	86.8
9	15	10.7	85.0
10	20	8.8	85.0
12	18	9.4	86.0
19	10	10.1	85.8
21	6	11.0	85.7

TOTAL	100	9.4	85.8

Amplification of the microsatellite markers was performed by multiplex PCR and the resulting fragments were separated according to size on an ABI PRISM 3700 (Applied Biosystems, Foster City, CA, USA). For quality control, each run included two CEPH samples (1331-01 and 1331-02) [17] and two water samples.

The Genotyper software v3.7 (Applied Biosystems) was used for allele calling. Marker order and intermarker distances were obtained from the Marshfield reference map [18]. *APOE *genotyping was performed at the respective research center.

### Statistical analysis

Family structures were verified through the Graphical relationship representation software (GRR) [19]. Mendelian errors were identified and allele frequencies for selected microsatellite markers were calculated including all available individuals using the MENDEL v8.0 software [20]. Allele frequencies were calculated for the total sample as well as for each of the analyzed subgroups: SWE, UK USA, *APOE ε4+ *(ARPs where both individuals have at least one *APOE ε4 *allele), and *APOE ε4- *(ARPs where neither individual has an *APOE ε4 *allele). File conversion was performed using Mega v4.0 [21].

Allele sharing multipoint LOD scores (MLS) and two-point LOD scores (TLS) were calculated for all groups using the Allegro v2.0 software [22]. As suggested by the software authors, an exponential model with scoring function S_pairs _and family weighting option "power: 0.5" was used. Significance levels of detected MLSs in the total sample and the analyzed subgroups were simulated through 1000 replications using the actual data set from the selected chromosomes.

## Results

In order to ensure high quality of the data included in the analysis, the GRR program was used on all available genotypes, including data from 270 microsatellite markers. This prompted us to exclude samples which displayed deviations from the expected average allele sharing between sibs or other family members. Having performed this quality check, 580 affected individuals from 261 pedigrees remained and were included in the analyses (Table 1).

The focus of this study was to further explore previously implicated linkage peaks from a genome scan by Myers *et al*. [10]. Both studies include samples from the UK and USA, whereas the NIMH sample used in Myers *et al*. was not included in the present scan, and samples from Sweden and Washington University were added. Using this modified sample collection, linkage to chromosomes 1, 9, 10, 12, 19 and 21 was analyzed in the whole sample and in subgroups based on sample origin or *APOE ε4 *status.

**Figure 1:**
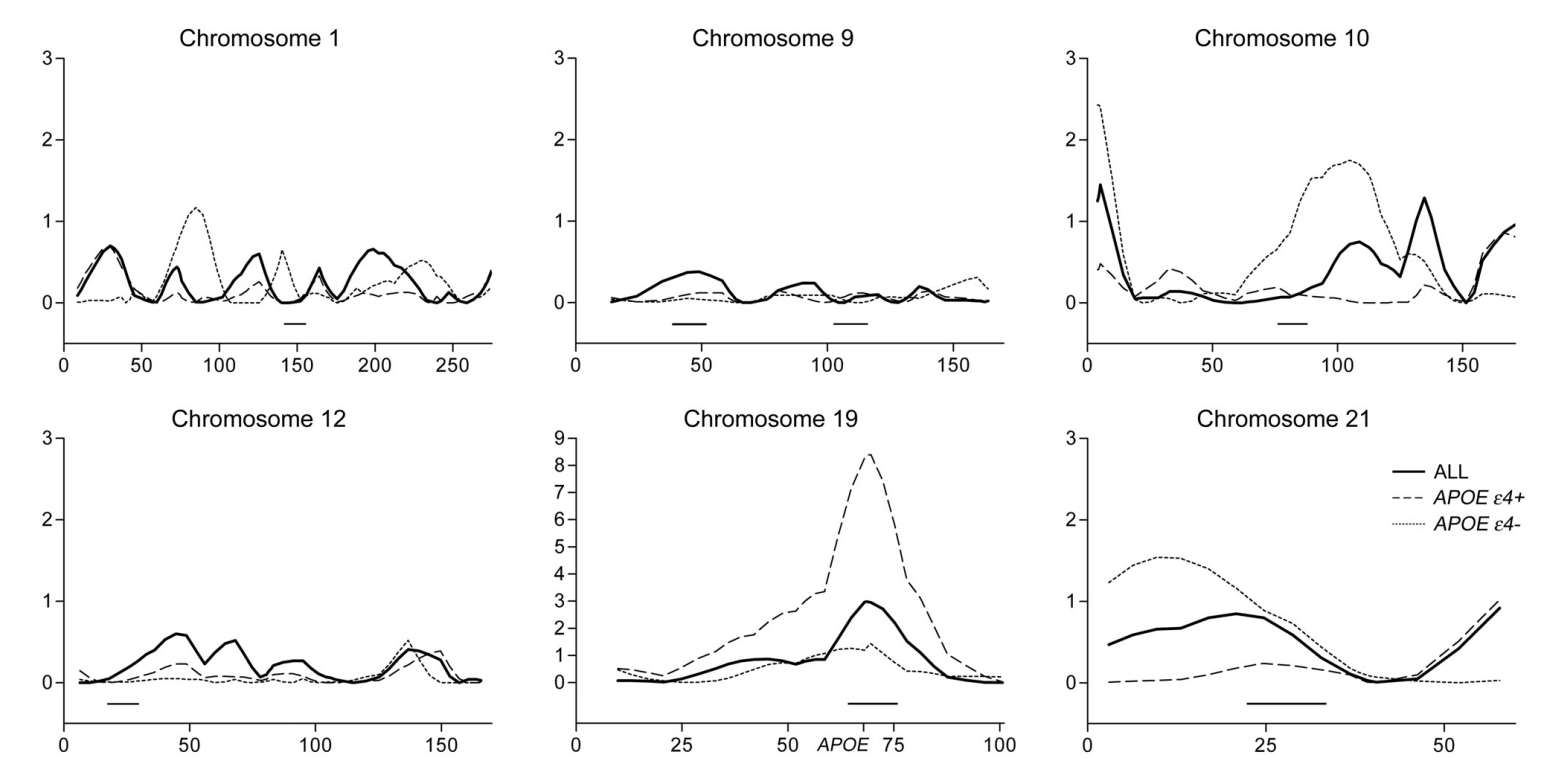
**Linkage results**. Linkage across selected chromosomes in the whole sample and the *APOE ε4+ *and *APOE ε4- *subgroups. Y-axes on all graphs represent MLS and X-axes are distance from pter in cM. MLS peaks (± 5 cM) from Myers *et al*. are indicated below the graphs.

Chromosome 19q13 demonstrated the highest MLS of 3.0 in the total sample, increasing to 8.3 in the *APOE ε4*+ subsample. Chromosome 1p36 revealed an MLS of 3.5 in the UK subgroup and chromosome 10p15 showed an MLS of 2.4 in the *APOE ε4- *subgroup. The region spanning chromosome 10q22-25 showed MLSs of 1.3, 1.8 and 1.9 in the total sample, the *APOE ε4- *and the USA subgroups, respectively (Table 3). Results from this linkage analysis have been depicted in Figure 1 with the positions of linkage peaks from Myers *et al*. denoted for comparison.

**Table 3:** Maximum MLS and (TLS) ≥1 from the present study

Chr region	Nearest marker (cM)	ALL	SWE	UK	USA	*APOE ε4+*	*APOE ε4-*
1p36	D1S3669 (37)	-	-	3.5^b ^(3.3^b^)	-	-	-
1p32	D1S3728 (85)	-	-	-	-	-	1.2
10p15	D10S1218 (5)	1.5^a^	-	-	1.5^a^	-	2.4^a ^(1.3)
10q22	D10S2327 (105)	-	-	-	-	-	1.8^a^
10q25	D10S1237 (135)	1.3 (1.1)	-	-	1.9^a ^(1.4)	-	-
10q26	D10S212 (171)	(1.2)	-	-	1.1	-	-
12q13	D12S398 (68)	(1.5^a^)	1.3 (1.4)	-	-	-	-
12q23	PAH (109)	-	1.1 (1.3)	-	-	-	-
12q24	D12S395 (137)	-	-	-	1.0	-	(1.6^a^)
12q24	D12S392 (166)	-	1.1	-	-	-	-
19p13	D19S586 (36)	-	1.7^a^	-	-	1.5	-
19q13	D19S178 (68)	3.0^b ^(2.5^b^)	1.9^a ^(1.6^a^)	-	1.1	8.3^b ^(7.3^b^)	1.2
21q21	D21S1437 (13)	-	-	-	-	-	1.5^a ^(1.6^a^)
21q22	D21S1446 (58)	(1.3)	-	-	-	1.0 (1.2)	-

Within each linkage region, the marker closest to the highest MLS has been denoted with its position in cM. Threshold levels were estimated from simulation analysis of the selected chromosomes. ^a^Suggestive and ^b^significant linkage for the respective linkage region and sample group according to Lander and Kruglyak [23].

Using data from the selected chromosomes, significance threshold levels were simulated for the total samples, as well as for the respective subgroups. After this simulation, linkage to chromosomes 19q13 in the total sample and the *APOE ε4*+ subgroup and to 1p36 in the UK subgroup are considered significant according to the definition by Lander and Kruglyak [23] (Table 3).

## Discussion

In the past decade there have been a number of attempts at identifying AD linkage regions using affected sib-pairs or extended families [8-14]. Although results have varied considerably and have sometimes been difficult to replicate, the most convincing linkage peaks have been reported from chromosomes 9, 10, 12, and 19.

In the present study, linkage peaks on chromosomes 1, 9, 10, 12, 19 and 21, previously implicated in a whole genome scan by Myers *et al*. have been further analyzed using a modified version of the original sample with an added collection of ARPs from Sweden and Washington University. We could detect significant linkage to chromosome 19q13 in the immediate vicinity of the *APOE *locus. This linkage peak was noticeably increased from the scan by Myers *et al*. and the Swedish sample contributed considerably to the improved linkage (Table 3). The influence of the *APOE *locus on AD has been correlated to a lower age at onset [4,24], as further demonstrated in our recent analysis of the chromosome 19 linkage [25]. Accordingly, linkage analysis of the NIMH cohort by Blacker *et al*. demonstrated the highest linkage to chromosome 19q13 in their subsample with earlier disease onset, whereas no linkage to this region was detected in their late onset sample [8]. The only other significant linkage found in the present study was to chromosome 1p36 in the UK subsample. However, this peak was neither detected in the other subgroups, nor in the total sample.

In the original whole genome scan by Myers *et al*., the most significant linkage peak was demonstrated on chromosome 10q21 (82 cM) in the whole sample. Blacker *et al*. also found linkage in the region, to chromosome 10q22 (92 cM) in their total collection of NIMH samples. In the present study, we could not detect linkage to chromosome 10q21, even though a suggestive linkage of MLS 1.8 was detected to chromosome 10q22 (105 cM) in the *APOE ε4- *sample. Although the sample size in the present study is smaller than in the study by Myers *et al*. (380 and 451 ASPs, respectively), the previous study by Kehoe *et al*. [9] using 292 ASPs and including overlapping samples with the Myers study also detected linkage to chromosome 10q21. This suggests that the absence of a linkage peak on chromosome 10q21 in the present study might be due to sample differences between the studies rather than sample size. However, we cannot completely exclude that our linkage to chromosome 10q22 in the *APOE ε4- *subsample coincides with the previously detected linkage to chromosome 10q21-22, although the positions of these peaks differ by 13-23 cM. Certain caution is also called for as the *APOE ε4- *subsample is rather limited in size (42 ARPs).

Inconsistent results between linkage studies might reflect heterogeneity in sample cohorts, including age at onset, ethnic background and diagnostic criteria. Our finding of significant linkage to chromosome 19q13, but to no other regions in the total sample in combination with the results presented by Blacker *et al*., suggests that finding significant linkage to both chromosome 19q13 and additional regions in the same sample is uncommon.

In the past few years, whole genome association studies have successfully identified susceptibility loci for a number of complex conditions. However, *APOE *is so far the only locus demonstrating strong association to AD [26-28]. Sample sizes have turned out to be crucial and sample collections including thousands of cases have been analyzed for association [29]. Increasing the number of samples in analyses also of ARPs would most likely be beneficial for the outcome and therefore further efforts to combine different sample collections should be made. It has been suggested that data from linkage analysis of affected sib-pairs could also be used to verify candidate susceptibility genes from association studies, since the frequency of a risk allele is expected to be higher in siblings sharing the locus than in population based cases [30].

## Conclusion

In this linkage study, we have analyzed a sample collection of AD ARPs from Sweden, the UK and the USA for linkage to chromosomes 1, 9, 10, 12, 19 and 21, implicated in the previous study by Myers *et al*. [10]. The highest linkage was detected on chromosome 19q13 close to the *APOE *gene. This linkage was extensively contributed by the Swedish samples, which has a lower average age at onset than the other subgroups. There was no evidence of the previously demonstrated linkage to chromosome 10q21 in the whole sample collection, and the relevance of the suggestive linkage within the *APOE ε4- *subgroup to chromosome 10q22 is somewhat uncertain due to the altered position of the peak and the restriction to this subgroup only. Our study demonstrates that chromosome 19q13 including *APOE*, at this point, is the only consistently linked locus for AD. This is also supported by genome wide association studies, demonstrating that *APOE *is the major susceptibility gene for AD [26]. Any additional susceptibility loci for AD are therefore likely to have much smaller effects, demanding very large sample sizes for detection.

## Competing interests

The authors declare that they have no competing interests.

## Authors' contributions

ESB carried out the genotyping, compiled data and participated in drafting the manuscript; VG carried out the statistical analyses; SA participated in the genotyping; MLH participated in creating the pedigree file; OA participated in the genotyping; A. Goate and JW participated in the design of the study; LL conceived the study and participated in its design and coordination; JH conceived the study, participated in its design and coordination, and revised the manuscript; FWDV participated in the genotyping and in designing and coordinating the study; A. Glaser participated in designing and coordinating the study and in drafting the manuscript. All authors read and approved the final manuscript.

## Pre-publication history

The pre-publication history for this paper can be accessed here:

http://www.biomedcentral.com/1471-2350/10/122/prepub

## Supplementary Material

Additional file 1 **Chromosomal positions of markers included in the scan**. This word DOC contains a table displaying chromosomal positions of markers included in the scan. Click here for file
