# Supplementary material for: Further analysis of previously implicated linkage regions for Alzheimer's disease in affected relative pairs
Source: BMC Med Genet. 2009 Dec 1;10:122. doi: 10.1186/1471-2350-10-122 (PMC2791756; doi:10.1186/1471-2350-10-122)
Supplement: Additional file 1 — Chromosomal positions of markers included in the scan. This word DOC contains a table displaying chromosomal positions of markers included in the scan. [file 1471-2350-10-122-S1.DOC]

| **Chromosome** | **Marker** | **Position (cM)** |
| --- | --- | --- |
| 1 | D1S2845 | 8.9 |
| 1 | D1S1612 | 16.2 |
| 1 | D1S1597 | 29.9 |
| 1 | D1S3669 | 37.1 |
| 1 | D1S552 | 45.3 |
| 1 | D1S1622 | 56.7 |
| 1 | D1S255 | 65.5 |
| 1 | D1S3721 | 72.6 |
| 1 | D1S2134 | 75.7 |
| 1 | D1S3728 | 89.5 |
| 1 | D1S1665 | 102.0 |
| 1 | D1S1728 | 109.0 |
| 1 | D1S551 | 113.7 |
| 1 | D1S1588 | 125.5 |
| 1 | D1S1631 | 136.9 |
| 1 | D1S3723 | 140.4 |
| 1 | D1S534 | 151.9 |
| 1 | D1S1653 | 164.1 |
| 1 | D1S1679 | 170.8 |
| 1 | D1S1677 | 175.6 |
| 1 | D1S1619 | 188.3 |
| 1 | D1S1589 | 192.1 |
| 1 | D1S518 | 202.2 |
| 1 | D1S1660 | 212.4 |
| 1 | D1S1248 | 226.0 |
| 1 | D1S2141 | 233.4 |
| 1 | D1S549 | 239.7 |
| 1 | D1S3462 | 247.2 |
| 1 | D1S235 | 254.6 |
| 1 | D1S547 | 267.5 |
| 1 | D1S1609 | 274.5 |
| 9 | D9S2169 | 14.2 |
| 9 | D9S1121 | 44.3 |
| 9 | D9S1118 | 58.3 |
| 9 | D9S301 | 66.3 |
| 9 | D9S1122 | 75.9 |
| 9 | D9S922 | 80.3 |
| 9 | D9S283 | 94.9 |
| 9 | D9S910 | 104.5 |
| 9 | D9S938 | 110.9 |
| 9 | D9S930 | 120.0 |
| 9 | D9S934 | 128.0 |
| 9 | D9S1825 | 136.5 |
| 9 | D9S2157 | 146.8 |
| 9 | D9S1826 | 159.6 |
| 9 | D9S1838 | 163.8 |
| 10 | D10S1435 | 4.0 |
| 10 | D10S1218 | 5.2 |
| 10 | D10S189 | 19.0 |
| 10 | D10S1412 | 28.3 |
| 10 | D10S2325 | 32.8 |
| 10 | D10S1423 | 46.2 |
| 10 | D10S1426 | 59.0 |
| 10 | D10S1208 | 63.3 |
| 10 | D10S1221 | 75.6 |
| 10 | D10S1225 | 80.8 |
| 10 | D10S1432 | 93.9 |
| 10 | D10S2327 | 100.9 |
| 10 | D10S2470 | 112.6 |
| 10 | D10S677 | 117.4 |
| 10 | D10S1239 | 125.0 |
| 10 | D10S1237 | 134.7 |
| 10 | D10S1230 | 142.8 |
| 10 | D10S1213 | 148.2 |
| 10 | D10S217 | 157.9 |
| 10 | D10S212 | 170.9 |
| 12 | D12S372 | 6.4 |
| 12 | D12S4912 | 18.0 |
| 12 | D12S391 | 26.2 |
| 12 | D12S373 | 36.1 |
| 12 | D12S1042 | 48.7 |
| 12 | D12S916 | 56.0 |
| 12 | D12S398 | 68.2 |
| 12 | D12S1294 | 78.0 |
| 12 | D12S375 | 80.5 |
| 12 | D12S1052 | 83.2 |
| 12 | D12S1064 | 95.0 |
| 12 | D12S1300 | 104.1 |
| 12 | PAH | 109.5 |
| 12 | D12S2070 | 125.3 |
| 12 | D12S395 | 136.8 |
| 12 | D12S2078 | 149.6 |
| 12 | D12S1045 | 160.7 |
| 12 | D12S392 | 165.7 |
| 19 | D19S591 | 9.8 |
| 19 | D19S1034 | 20.8 |
| 19 | D19S586 | 32.9 |
| 19 | D19S714 | 42.0 |
| 19 | D19S433 | 51.9 |
| 19 | D19S245 | 58.7 |
| 19 | D19S178 | 68.1 |
| 19 | *APOE* | 69.6 |
| 19 | D19S246 | 78.1 |
| 19 | D19S589 | 87.7 |
| 19 | D19S254 | 100.6 |
| 21 | D21S1432 | 3.0 |
| 21 | D21S1437 | 13.1 |
| 21 | D21S2052 | 24.7 |
| 21 | D21S1440 | 36.8 |
| 21 | D21S2055 | 40.5 |
| 21 | D21S1446 | 57.8 |
